# Isolation, Preliminary Structural Insights, Characterization, and Antioxidant Potential of a New High-Molecular Weight Complex Phenolic Polymer Developed from Olive Mill Wastewater

**DOI:** 10.3390/antiox14070791

**Published:** 2025-06-27

**Authors:** Antonio Lama-Muñoz, Alejandra Bermúdez-Oria, Fátima Rubio-Senent, Guillermo Rodríguez-Gutiérrez, África Fernández-Prior, Juan Fernández-Bolaños

**Affiliations:** Departamento de Fitoquímica de los Alimentos, Instituto de la Grasa (CSIC), Campus de la Universidad Pablo de Olavide, Edificio 46, Ctra. de Utrera km 1, 41013 Sevilla, Spain; aleberori@ig.csic.es (A.B.-O.); f.r.senent@csic.es (F.R.-S.); guirogu@ig.csic.es (G.R.-G.); mafprior@ig.csic.es (Á.F.-P.); j.fb.g@csic.es (J.F.-B.)

**Keywords:** antioxidant capacity, ATR-FTIR analysis, column chromatography, complex phenols, gel filtration, natural phenolic polymers, olive mill wastewater, structural characterization

## Abstract

Olive mill wastewater (OMW), a byproduct of the olive oil industry, is a potential source of natural bioactive phenolic polymers. In this work, a column chromatography technique was used for the isolation of a new complex polymer (named OMW-2000XAD) from OMW via fractionation on Amberlite^®^ XAD16 resin. The developed procedure was simple and proved to be reproducible using OMW from two different sources. OMW-2000XAD was further characterized by elemental, glycosidic, and amino acid composition analysis, as well as spectroscopic techniques. The polymer’s molecular size, which was estimated via gel filtration chromatography, was 1960 kDa, which is significantly larger than other high-molecular weight fractions previously isolated from OMW or other agro-industrial wastes. OMW-2000XAD was mainly composed of phenolic compounds (89.8%). It also contained polysaccharides (16.1%) and proteins (10.3%), with glucose (12.25%) and cysteine (1.71%) being the most abundant sugar and amino acid, respectively, as well as metals (1.29%, primarily potassium). However, due to its low solubility, complexity, and heterogeneous composition, it was not possible to identify all phenolic compounds or elucidate a definitive structure via MS, FTIR, and NMR. OMW-2000XAD exhibited strong radical scavenging antioxidant capacity (ABTS^•+^, DPPH^•^ and peroxyl radicals), with results up to 7415 µmol Trolox equivalent/mol (ORAC method), but showed no antiproliferative effects, highlighting the need for further research.

## 1. Introduction

In Spain, the two-phase decanter system is used in nearly all olive oil mills. However, in other Mediterranean countries (including Italy, Greece, Tunisia, Turkey, Morocco, Portugal, and Syria), this technology is still not widely used. Thus, it is estimated that between 10 and 30 million m^3^ of olive mill wastewater (OMW) is produced annually worldwide [1]. Furthermore, it is likely that in the future, Spanish olive pomace oil extraction plants will return to a three-phase system for processing olive pomace (*alperujo*), thereby generating more OMW. Some major plants already use this system, as it results in a lower moisture content in the olive pomace.

The physicochemical composition of OMW basically consists of around 83–94% water, 4–16% organic compounds (phenols, proteins, sugars, etc.), and 0.4–2.5% inorganic salts [2]. OMW contains high concentrations of phenolic compounds, comprising 98% of the phenols in olive fruit and 2–15% of the organic fraction. Concentrations were found to vary from about 0.5 g/L to 24 g/L [3]. More than 80 individual phenolic compounds distributed across a dozen different chemical classes have been identified in OMW, depending on factors such as olive variety, climatic conditions, or the olive oil extraction system [4,5]; however, OMW also contains polymeric phenols, such as lignin, tannins, and pigments identified as catechol–melanin macromolecules [6], as well as other high-molecular weight complex phenolic fractions that are responsible for its characteristic dark color. OMW reacts easily with atmospheric oxygen. Particularly, phenolic compounds are catalytically oxidized by polyphenol oxidases and peroxidases to colored *o*-quinones that further undergo non-enzymatic secondary condensation reactions with the original phenols, resulting in various polymers. The large number of compounds contained in OMW and their varied structural diversity unavoidably lead to interactions (covalent or not) with other molecules and/or macromolecules [7]. These *o*-quinones are highly reactive electrophiles that can also form adducts with amino acids and proteins [8] or react with sugars or polysaccharides (glycosyl derivatives). The interaction among polyphenols, proteins, and polysaccharides may result in complex macromolecular structures [9], high-molecular weight polymeric substances, or brown pigments that are difficult to characterize.

Currently, the economic value of OMW is certainly low; however, there is an increasingly close collaborative relationship between the olive oil sector and the sphere of scientific research to improve management, reduce disposal costs and environmental impacts, and achieve more profitable valorization. This collaboration focuses on developing strategies [10,11], technical innovations [12], and finding solutions to the disposal problems of a pollutant byproduct (BDO, 35–110 g/L; COD, up to 400 g O_2_/L) [3,13] and converting it into a raw material source of high-value-added products to create a change in the negative perception of this liquid waste (from waste to resource).

Among the possible alternatives, obtaining complex phenolic polymers is a potential valorization option for OMW due to their various interesting applications. Natural phenolic polymers (NPPs) obtained from agro-food wastes and byproducts can be utilized for the remediation and treatment of contaminated waters and/or wastewater due to their excellent sorption capacity for drugs, inorganic anions, and organic dyes [14]. In line with this, OMW phenolic polymers were shown to be efficient sorbents of heavy metals, herbicides, and polycyclic aromatic hydrocarbons [15,16]. Several naturally occurring phenolic polymers have been explored and/or tested in active packaging and film stabilization for food applications [17]. For example, lignin and tannins have been demonstrated to be effective at improving mechanical properties (such as viscoelasticity and tensility) and providing barrier and protective functions (oxygen permeability, UV resistance, thermal stability, antimicrobial, antioxidant, etc.) when incorporated into biodegradable plastics (such as PHB, PLA, and alginate), starch-based films, and polyethylene or polypropylene. NPPs, including those aggregated or conjugated with polysaccharides and proteins, have also been investigated for various medical and health-related applications. These include use as promoter additives for cell differentiation in tissue engineering [18], anticoagulants [19], and in vivo hypolipidemic agents [20].

Existing studies on complex polymers derived from OMW are limited to a few authors. Moreover, the structural characterization of these complexes and polymeric phenolic fractions remains scarcely elucidated. Capasso and co-workers [21] are among the first researchers to address the recovery and characterization of a polymeric organic fraction from OMW. They isolated the fraction via precipitation with cold methanol, so-called “polymerin”, composed of polysaccharides, melanin, and proteins strongly conjugated to each other by a combination of supramolecular interactions. This polymerin, with a relative molecular size between 2 and 500 kDa, has demonstrated its potential utilization in environmental technologies and industry [22] and as a bioamendment in agriculture [23], since the degree of similarity between it and humic acids is remarkable. Polymerin has also been detected in dry olive pomace [24]. Cardinali and co-workers [25] obtained several fractions containing unidentified phenolic polymers (600–5000 Da) via the ultrafiltration of OMW. The data reported indicate that these fractions proved to be more efficient antioxidants than hydroxytyrosol. In a previous work [26], we studied a polymeric phenolic fraction (PPF), which was extracted with ethyl acetate from steam-treated alperujo hydrolysates. It was different from that recovered by Capasso and co-workers [21] because of its physical properties (for example, solubility) and its quantitative composition (mostly phenolic compounds); however, it had a characteristic HPLC-DAD chromatographic profile very similar to those described previously for other phenolic polymers derived from OMW [24,27]. The PPF showed an excellent sorption–desorption capacity for phenolic compounds and activity against lipidic oxidation comparable with those of the best antioxidants, such as hydroxytyrosol and vitamin E. More recently, Khemakhem and co-workers [28,29] have isolated a protein–polyphenol polymer complex (5–190 kDa) from OMW via precipitation with ammonium sulfate and characterization via spectroscopic analyses that has similar characteristics to melanin and humic substances and surfactant properties.

During an exploratory chromatographic fractionation of OMW using XAD16 resin to obtain low-molecular weight organic and phenolic acids, an unexpected high molecular compound eluting with ethanol was detected. Preliminary analyses showed the complexity and mainly phenolic nature of this high-molecular weight substance. With all this background, the main aim of the work was to isolate this new large-molecular size complex phenolic material by establishing a simple recovery method via column chromatography. This separation technique has not been used previously for isolating valuable OMW polymers. The selection of the methodology is essential for the economic and sustainable valorization of OMW. This study also aims to characterize the new isolated material using spectroscopic analysis and assess its potential antioxidant and antiproliferative activities.

## 2. Materials and Methods

Two batches of Picual olives at different ripeness stages were collected from two locations; the first was harvested in the Spanish province of Granada, and the second one was harvested in an experimental olive orchard property of the Instituto de la Grasa (CSIC) in the Spanish province of Seville. Thus, two different OMW samples (OMW1 and OMW2, respectively) were extracted on a laboratory scale via the ABENCOR^®^ System (MC2 Ingeniería y Sistemas, S.L., Seville, Spain), which accurately replicates the industrial olive oil extraction process. OMW samples were separated from the oily phase via decantation and then stored at 4 °C until the start of the experiment. A total of 3.6 L of OMW1 and 1.8 L of OMW2 were obtained.

### 2.1. Isolation and Purification of the OMW Complex Phenolic Polymer

The OMW fractionation was performed using AMBERLITE^®^ XAD16 resin (Rohm and Hass, Philadelphia, PA, USA). AMBERLITE^®^ XAD16 is useful for the adsorption of organic substances from aqueous systems and, specifically, for the separation of large organic molecules. The resin (770 g) was packed into a 4 cm-diameter (ID) glass chromatography column with a 60 cm bed height. Prior to fractionation, the OMW1 sample was filtered, concentrated 3-fold (from 3.6 L to a final volume of 1.2 L), and defatted with hexane. A 600 mL aliquot of the concentrated OMW was transferred onto the column. After all the OMW aliquot entered the column, eighteen 250 mL fractions were collected via elution with distilled water at a flow rate of 15 mL/min. Thus, the column was washed to remove all individual or unbound compounds. Next, the complex phenolic polymer was obtained by eluting three more 250-milliliter fractions with 96% ethanol, numbered as 19, 20, and 21. The complex eluted in fraction 21 was not detected in subsequent fractions, as confirmed via an HPLC-DAD analysis performed according to the procedure described in Section 2.2 (only fraction 21 showed a broad UV-absorbing peak). We named the complex polymer OMW-2000XAD. Finally, the OMW-2000XAD fraction (250 mL) was dialyzed to remove impurities and lower-molecular weight solutes using 12,000 molecular weight cut-off cellulose membrane dialysis tubing (D-9402, Sigma Chemical Co., St. Louis, MO, USA). Dialysis tubing was transferred to a 2 L beaker containing double-distilled water. After 24 h of incubation with continuous gentle agitation, the solution was replaced with fresh double-distilled water. This step was repeated several times until the external solution became colorless (a total volume of 5 L of water was required). The OMW-2000XAD dialyzed fraction (non-permeating fraction) was then lyophilized, yielding 4.215 g of dry residue. A similar result was achieved when the other 600 mL aliquot was fractionated, yielding 4.240 g of the complex after lyophilization. To verify repetitiveness, the isolation procedure was reproduced with the second OMW sample. OMW2 was also concentrated 3-fold up to a final volume of 0.6 L. This trial with OMW2 also yielded a similar amount of the complex (4.197 g), supporting the robustness of the methodology. The analyses performed on the complexes obtained from OMW1 and OMW2 to compare the identity of both samples (HPLC-DAD chromatographic profile, estimation of molecular weight via gel filtration chromatography, determination of antioxidant capacity via the DPPH method, amino acid composition, and phenolic profile after alkaline hydrolysis) did not reveal significant differences between them. For this reason, all recovered complexes from the three trials were pooled and treated as a single sample. Therefore, the final yield factor was 2.34 g OMW-2000XAD/L OMW or 0.23% (*w*/*v*).

#### Water Solubility

The water solubility of OMW-2000 XAD was gravimetrically determined in triplicate. In a stepwise procedure, increasing amounts of complex were added at 25 °C to 15 mL of distilled water in a conical flask. After each addition, the solution was shaken in a vortex for 1 min, allowing for equilibration, and then visually checked for any undissolved part of the sample until a saturated solution was obtained. The saturated solution was centrifuged and filtered, and 10 mL of the filtrate was pipetted out into a pre-weighed round-bottom flask to be lyophilized. The residue was stored in a desiccator until a constant weight was reached. The solubility calculation was as follows: (weight of the round-bottom flask + dry residue) − (weight of the empty round-bottom flask) g of OMW-2000XAD requires [(weight of the round-bottom flask + 10 mL solution) − (weight of the round-bottom flask + dry residue)/density of water at 25 °C] mL of water. Solubility was expressed quantitatively in terms of percentage (*w*/*v*).

### 2.2. HPLC-DAD Chromatographic Analysis

An aqueous solution of lyophilized OMW-2000XAD (16 g/L) was analyzed via HPLC using an HP Series 1100 liquid chromatograph (Hewlett Packard, Palo Alto, CA, USA) combined with DAD monitoring. A Kinetex EVO C18 LC column (5 μm, 100 Å, 250 × 4.6 mm) (Phenomenex, Torrance, CA, USA) was utilized for separation at 25 °C at a flow rate of 1 mL/min. A 20 µL sample volume was injected for the analysis. The composition of mobile phases was as follows: mobile phase A consisted of 0.01% trifluoroacetic acid in Milli-Q water, and mobile phase B consisted of 100% acetonitrile. Chromatographic separation was carried out in gradient mode as follows: 0 min, 5% B; 0–30 min, from 5% to 25% B; 30–45 min, from 25% to 50% B; 45–47 min, from 50% to 100% B; 47–50 min from 100% to 25% B; 50–52 min, from 25% to 5% B, and finally, 52–55 min, 5% B. Detection was performed at 280 nm.

### 2.3. Multi-Elemental Composition and Protein Content

For the analysis of carbon and nitrogen, a LECO CHN828 Series analyzer instrument (LECO Corporation, St. Joseph, MI, USA) was used. This equipment used a combustion technique with a vertical quartz furnace to determine the carbon and nitrogen contents. OMW-2000XAD was automatically weighed into a tin capsule and loaded into the analyzer. The sample first entered a sealed purge chamber to remove atmospheric gas. After purging, OMW-2000XAD was transferred to the furnace, where it was combusted in an oxygen-rich environment for rapid oxidation. Gases from the furnace were analyzed for carbon (CO_2_) and nitrogen (N_2_). The protein content was then calculated as % nitrogen using a conversion factor of 6.25, which is a common practice for estimating protein content. An acidic amino acid composition analysis and tryptophan content measurement were carried out according to the procedure described by Rodríguez-Martín and co-workers [30].

For the multielement analysis, 50 mg of OMW-2000XAD, which was accurately weighed, was subjected to a microwave-assisted acid digestion according to the EPA method 3051A. A MARS 1™ microwave digestion system (CEM Corporation, Matthews, NC, USA) was used. The digested samples, in duplicate, were then filtered through 0.22 μm and conveniently diluted with aqua regia prior to analysis via inductively coupled plasma mass spectrometry (ICP-MS) on an Agilent 7800 Quadrupole ICP-MS (Agilent, Santa Clara, CA, USA) working in He gas mode. Calibration solutions covering a range from 0 to 500 µg/L were prepared from ICP-MS grade multi-element standards in 1% HNO_3_. The operating conditions of ICP-MS were as follows: plasma gas flow (Ar), 15 L/min; temperature, 8000 K; RF power, 1.6 kW; pump rate, 13 rpm; replicates, 5; scan per replicate, 10; and spray chamber temperature, 2 °C. The results are based on independently prepared triplicates and expressed as means ± the standard deviation.

### 2.4. Estimation of the Total Phenolic Content (TPC)

The Folin–Ciocalteu method [31] was used for the analysis of total phenols, but with some modifications. A total of 100 μL of conveniently diluted OMW-2000XAD (0.625 g/L) in 50% (*v*/*v*) methanol–water, gallic acid (0–5 μg) as standard, and 50% (*v*/*v*) methanol–water blanks were added to triplicate 5 mL test tubes. Next, 200 μL of 0.2 M Folin–Ciocalteu reagent and 800 μL of 0.7 M sodium carbonate were added into each test tube, vortexed thoroughly, and incubated at room temperature for 10 min. Finally, 200 μL from each assay tube was transferred to a 96-well microplate, and the absorbance was read at 655 nm using an iMark™ microplate absorbance reader (Bio-Rad Laboratories, Inc., Hercules, CA, USA). The results were expressed as means of three replicates analyzed separately (µmol gallic acid equivalents/g OMW-2000XAD) ± the standard deviation.

### 2.5. Determination of Sugars via Gas Chromatography (Alditol Acetates)

A total of 10 mg of OMW-2000XAD containing approx. 300 μg of sugars (determined via the anthrone method) were placed into a glass tube in triplicate. A total of 0.5 mL of 2 N trifluoroacetic acid (TFA) and 50 μg of inositol as the internal standard were added. The tubes were incubated using a heating block for 1 h at 121 °C. After this, the hydrolysates were evaporated to dryness in a stream of air and washed three times with 1 mL of MeOH. Next, 400 μL of 2 N NH_4_OH containing 10 mg/mL NaBH_4_ was added to reduce the monosaccharides to alditols and incubated at 40 °C for 1 h. Later, glacial acetic acid was dropped into each tube to remove the excess NaBH_4_. After that, the tubes were washed three times with glacial acetic acid–methanol (1:9, *v*/*v*) and then with methanol. The reaction mixture was acetylated by adding 120 μL of methylimidazole and 600 μL of acetic anhydride and incubating for 10 min at room temperature. At last, 5 mL of distilled water was added to each tube to destroy the excess acetic anhydride, and then, 2 mL of dichloromethane was used to extract the alditol acetates. The separation and quantification of the alditol acetates were performed using a gas chromatograph (Hewlett Packard 6890 Series) fitted with a FI detector and a SP^®^-2330 capillary column (L × I.D. 30 m × 0.25 mm, d_f_ 0.20 μm) (Merck KGaA, Darmstadt, Germany). The chromatographic conditions were described previously by Jaramillo-Carmona and co-workers [32]. The results are expressed as means of three replicates analyzed separately ± the standard deviation.

### 2.6. Estimation of Molecular Size via Gel Permeation Chromatography (GPC)

The molecular size estimation of the OMW-2000XAD complex polymer was accomplished by using TSKgel G3000PWXL and GMPWXL (both 7.8 mm ID × 30 cm) columns (Tosoh Bioscience LLC, Tokyo, Japan) operating in series at a flow rate of 0.8 mL/min. The void volume and total volume were measured using solutions of Blue Dextran 2000 (2000 kDa) (GE HealthCare, Chicago, IL, USA) and glucose (180 Da), respectively. Dextrans from *Leuconostoc* spp. with average molecular weights of 500 kDa, 110 kDa, 70 kDa, 40 kDa, and 6 kDa (Fluka Chemie AG, Buchs, Switzerland), were used as analytical standards to calibrate the molecular weight of OMW-2000XAD. Refractive index detection was used in this analysis.

### 2.7. Attenuated Total Reflectance (ATR)–Fourier-Transform Infrared (FTIR) Spectroscopy

The FTIR spectrum was recorded using an INVENIO X spectrometer (Bruker Optics GmbH & Co. KG, Ettlingen, Germany) fitted with a mid-infrared (MIR) source, a KBr beam splitter, and a DLaTGS pyroelectric detector. A total of 1 mg of lyophilized OMW-2000XAD was directly placed on the diamond ATR crystal surface. The spectrum was recorded in the range of 4000–400 cm^−1^ and averaged over 32 scans (scan speed of 20 kHz) at a resolution of 4 cm^−1^. This analysis was performed by the Research, Technology and Innovation Centre (CITIUS) at the University of Seville (Spain).

### 2.8. Nuclear Magnetic Resonance Spectroscopic Analysis

Nuclear Magnetic Resonance (NMR) spectra were recorded in water. Analyses were performed with a Bruker Avance III 500 MHz NMR spectrometer equipped with a 5 mm ^1^H/^13^C/^15^N inverse detection probe with a Z gradient, and lock ^2^H. ^1^H and ^13^C chemical shifts were referenced to DSS (δ = 0 ppm). Longer experiments, such as ^13^C{^1^H} and 2D HSQC spectra, were obtained after 5 days of acquisition. NMR spectra were processed with TopSpin^®^ software (version 3.2.0).

### 2.9. Acid and Basic Hydrolysis and Analysis of Products via Mass Spectrometry

In order to investigate the composition and structure of OMW-2000XAD, acid and alkali-catalyzed degradations with 6 M HCl (at 110 °C for 24 h) and 4 M NaOH (at 120 °C for 4 h) were carried out in an atmosphere of nitrogen. Then, the produced hydrolysates were neutralized and extracted with ethyl acetate. Analyses of the hydrolysates (both neutralized and not neutralized) and the resulting ethyl acetate extracts were performed using mass spectrometry to characterize the nature of the constitutive compounds that are found in the OMW-2000XAD complex.

The hydrolysates were separated in a DIONEX UltiMate 3000 RS UHPLC^+^ focused (Thermo Scientific, Waltham, MA, USA) equipped with a quaternary pump, an autosampler, and a photodiode array detection (DAD) system. Chromatographic separation was performed on a mediterranea™ sea18 column (20 cm × 4.6 mm I.D., 3 µm particle size) (Teknokroma, Barcelona, Spain) at 30 °C. An online pre-column Ultraguard™ sea18 was added to protect the column. The mobile phases consisted of 0.1% (*v*/*v*) formic acid in water (A) and acetonitrile (B). The proportion of B increased from 5% to 25% in 30 min, to 50% in 15 min, to 100% in 2 min, and to 25% for 3 min. The initial conditions were reached in 2 min, and the equilibrium time was 3 min. The injection volume was 20 µL, and the flow rate was 1 mL/min. The DAD system was set to scan from 190 to 400 nm, and 280 and 340 nm were used as the detection wavelengths for the compounds. A split post-column of 0.4 mL/min was introduced directly on the mass spectrometer for detection. Mass spectrometry was performed on a micrOTOF-Q II^TM^ Ultra-High Resolution Qq-Time-of-Flight mass spectrometer (Bruker Daltonics, Bremen, Germany) equipped with an electrospray ionization (ESI) source operating in negative ion mode. The ESI source conditions were as follows: drying gas flow rate 8 L/min; drying gas temperature, 200 °C; nebulizer pressure, 1.2 bar; capillary voltage, 3.5 kV. Mass spectra were acquired in full scan mode using a scan range of *m*/*z* 50–1500 Da. Instrument control and data evaluation were performed with Bruker Daltonics HyStar 3.2 and Bruker Daltonics DataAnalysis 4.2, respectively.

### 2.10. Antioxidant Capacity Assays (DPPH, FRAP, ORAC, and TEAC)

The 2,2-diphenyl-1-picrylhydrazyl radical scavenging capacity (DPPH) assay, the ferric ion reducing antioxidant power (FRAP) assay, and Trolox equivalent antioxidant capacity (TEAC) were carried out as described by Lama-Muñoz and co-workers [33].

On the other hand, an oxygen radical absorbance capacity (ORAC) assay was run according to the following procedure: 25 μL of adequately diluted OMW-2000XAD sample (0.025 g/L), Trolox standard solutions (ranging from 10 to 140 μM), and 10 mM phosphate buffer (Na_2_HPO_4_·12 H_2_O/NaH_2_PO_4_·H_2_O), as a blank, were added in triplicate to 96-well microplates. To all wells, 150 μL of 1 μM fluorescein freshly prepared solution was added. A Fluoroskan Ascent^®^ (Thermo Fisher Scientific, Waltham, MA, USA) microplate fluorometer reader was used for measurements. The microplate was then allowed to equilibrate by incubating for 15 min at 37 °C. The reaction was initiated by the addition of 25 μL of 250 mM 2,2′-azobis (2-amidinopropane) dihydrochloride (AAPH), which was also dissolved in phosphate buffer. Excitation was performed at 485 nm, and emission was measured at 538 nm. The fluorescence of each well was then measured every 5 min, for a total analysis time of 90 min. As the reaction progressed, fluorescein (FL) was consumed, and its intensity decreased. In the presence of antioxidants, FL decay is inhibited [34]. ORAC values were obtained by calculating of the area under the kinetic curve [relative fluorescence intensity (f_i_) vs. time (i = 0, 5, 10, …, 90 min)] (A = (0.5 + f_5_/f_0_ + f_10_/f_0_ + … + f_90_/f_0_)) and plotting it versus concentration.

All results were expressed as means of three replicates analyzed separately (µmol Trolox equivalents/g OMW-2000XAD) ± the standard deviation.

### 2.11. Anti-Proliferative Activity Assays

#### 2.11.1. Caco-2 Cell Culture

Caco-2 cells were maintained at 37 °C and 5% CO_2_ in Dulbecco’s Modified Eagle Medium (DMEM), which contained 1 g/L of glucose, 0.11 g/L of pyruvate, and 0.58 g/L of glutamine. The medium was supplemented with 10% heat-inactivated fetal bovine serum (at 56 °C for 30 min), 1% non-essential amino acids, 100 U/mL of penicillin, and 100 µg/mL of streptomycin. Cells were subcultured weekly using trypsin-EDTA, and the culture medium was refreshed once between passages.

#### 2.11.2. Caco-2 Cell Line Proliferation Assay with OMW-2000XAD

Experiments were performed using conventional incubation conditions. The culture medium was substituted with lyophilized OMW-2000XAD, which was previously dissolved in Hank’s Balanced Salt Solution (HBSS) at a concentration of 100 mg/mL and further diluted with medium culture as needed. Caco-2 cells were seeded in 96-well microplates at a density of 4 × 10^4^ cells per well in 50 μL of medium. Equal volumes of OMW-2000XAD solution were added to achieve final concentrations ranging from 0.08 to 10 g/L. Cells were incubated for up to 3 days and assessed.

In a parallel set of experiments, the assay was additionally conducted on confluent Caco-2 cells, which were seeded in 96-well microplates at a higher density of 14 × 10^4^ cells per well in 100 μL of medium. These cells were allowed to proliferate for two days before being exposed to increasing concentrations of OMW-2000XAD (0.31–10 g/L). Confluent Caco-2 cells were evaluated on days 3, 5, 9, and 11.

The neutral red assay was used to assess the cell viability of Caco-2 cells in both experiments, as described by Borenfreund and Puerner [35]. Cells in 96-well microplates were incubated at 37 °C with fresh culture medium containing neutral red dye (50 μg/mL). After 30 min, cells were washed with HBSS and then solubilized with 75 μL of a glacial acetic acid solution (1% *v*/*v*) in 50% (*v*/*v*) ethanol in water. Absorbance was then measured at 540 nm on a microtitre plate reader.

### 2.12. Statistical Analysis

All experiments were performed in triplicate (three replicates analysed separately). Data are expressed as mean ± standard error (*n* = 3). Excel software (Microsoft Corp., Redmond, WA, USA) was used to perform Fisher’s Least Significant Difference (LSD) test in one-way ANOVA. Statistical significance was evaluated when applicable.

## 3. Results and Discussion

### 3.1. Chemical Composition Analysis

**Figure 1:**
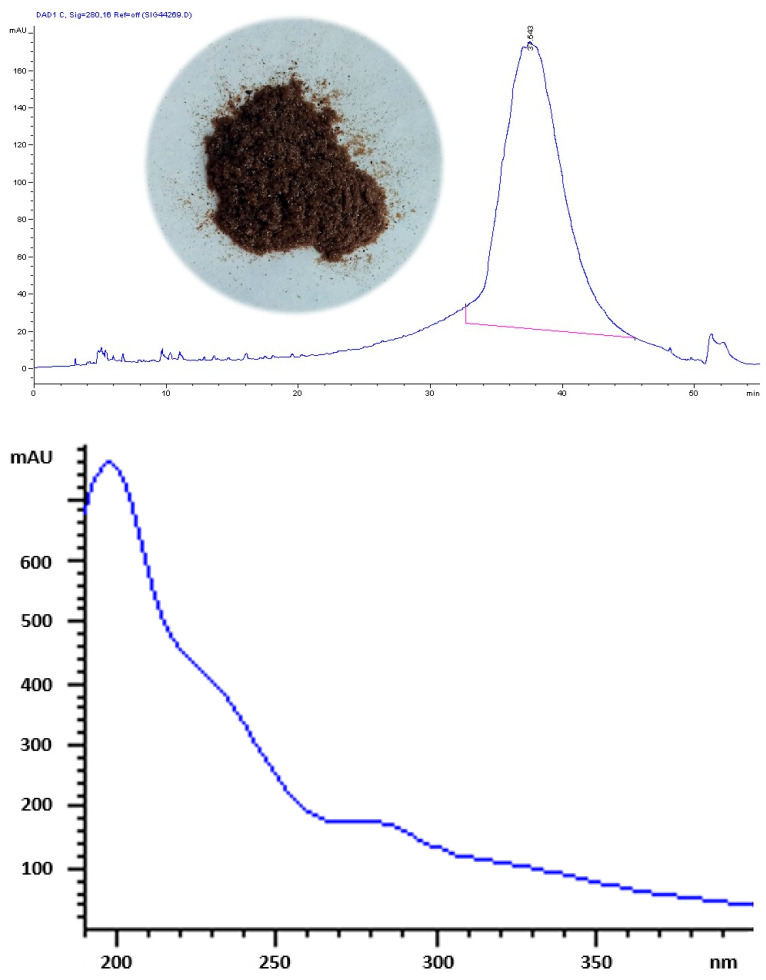
Representative HPLC chromatogram at 280 nm (**above**) and UV spectrum (**below**) of the complex OMW-2000XAD studied herein. Superimposed on the chromatogram, a photograph of the appearance of OMW-2000XAD is included.

As shown in Figure 1, OMW-2000XAD displays a characteristic HPLC-UV chromatographic profile with a broad peak signal in the 34–46 min retention time range. This profile was also observed for other complex polymeric fractions from olive mill wastes [24,26]. Likewise, the UV absorption spectrum closely resembles those of other complex polymers isolated from OMW [28,36], including other polyphenolic–polysaccharide complexes with different plant origins [20], and it stands out for its featureless shape. The absorbance of OMW-2000XAD decreases rapidly until it reaches 260 nm and creates a slight shoulder around 230 nm. Approximately in the range between 260 nm and 290 nm, the slope break of the spectrum is produced, which is attributable to the presence of unsaturation in the aromatic structures of phenolic components, quinones, or oxidation products [37], as well as proteins. Finally, the signal gradually decreases toward the end of the spectrum.

Once lyophilized, OMW-2000XAD is chemically very stable and non-hygroscopic. Its physical appearance and composition remain unchanged, even when stored at room temperature or exposed to the atmosphere. The solubility of OMW-2000XAD in water at 25 °C was determined by saturation until the appearance of insoluble precipitate. The solubility was 5.2 g/100 mL. It is also soluble in ethanol/methanol aqueous solutions (50%, *v*/*v*), but in a lower ratio. OMW-2000XAD is insoluble in most organic solvents (including acetone, acetonitrile, chloroform, dichloromethane, ethanol, ethyl acetate, hexane, methanol, and tetrahydrofuran), except for DMSO. In this aspect, OMW-2000XAD differs from a polymeric phenolic fraction previously isolated by the authors that was completely soluble in ethanol and only slightly soluble in water [26]. With regards to the polymerin [21] and OMWW-ASP [28], a comparison cannot be made because these researchers do not report water solubility data but only indicate “very soluble polymerin” and “a water-soluble polymer complex”, respectively.

OMW-2000XAD was shown to be a complex natural substance that is mainly composed of phenols (89.8%) linked to polysaccharides (16.1%) and proteins (10.3%; calculated on the basis of nitrogen content, 1.64%) (Table 1). The discrepancy in the value of TPC is because the Folin–Ciocalteu reagent is unspecific and can react with other molecules, such as sugars and proteins [38], present in OMW-2000XAD, thereby leading to the overestimation of the content of phenols. The phenolic content of OMW-2000XAD differs significantly compared with those of other complex fractions isolated from OMW, which was much higher than that of polymerin recovered by Capasso and co-workers [21] (13.3%) or that of the polymer complex (OMWW-ASP) separated by Khemakhem and co-workers [28] (1.6%). This difference can be explained by the fact that both researchers and their colleagues used selective precipitation via cold methanol, which favors the precipitation of carbohydrates (polymerin contains 52.4%), and ammonium sulphate, which favors the precipitation of proteins (OMWW-ASP has 31.1%), while OMW-2000XAD was isolated using column chromatography.

The total measured concentration of metals in OMW-2000XAD was 1.29% (*w*/*w*). However, polymerin has an 11.06% metal concentration. The metal composition found in OMW-2000XAD includes, in decreasing order, K (0.73%, *w*/*w*), Ca (0.34%), and Mg (0.13%), and to a lesser extent, Na, Fe, and Cu have the same concentrations (0.03%). Other elements that occur in smaller amounts include Mn and Zn. The K element reported in weight percentage is also the major metal in the polymerin.

The quantitative assay of the neutral sugars produced via the hydrolysis of OMW-2000XAD revealed the presence of polysaccharides, with a glucose percentage of 12.25%, followed by arabinose (1.11%), rhamnose (0.91%), galactose (0.76%), mannose (0.58%), and xylose (0.49%) (Table 2). This quantitative order contrasts with Capasso and co-workers’ results, who found that the main sugar in polymerin was arabinose (20.9%). However, conversely, Nadour and co-workers [39] have also reported that glucose was the main monosaccharide in OMW, followed by the rest of the sugars in the same order measured herein. This difference can be explained by the fact that the procedure used is distinct from those of other authors. On the other hand, the colorimetric analysis of uronic acids yielded a negative indication of their presence in OMW-2000XAD.

The amino acid composition of OMW-2000XAD is also presented in Table 2. It is similar to that found in the polymerin. Glutamic acid (1.27%), aspartic acid (1.13%), glycine (1.08), serine (0.75%), and alanine (0.58%) showed higher contents; these are also among the major amino acids in polymerin. However, there is an important difference: the content of cysteine is 1.71%, while it is a minor amino acid in polymerin (0.1%).

### 3.2. Gel Filtration Chromatography

**Figure 2:**
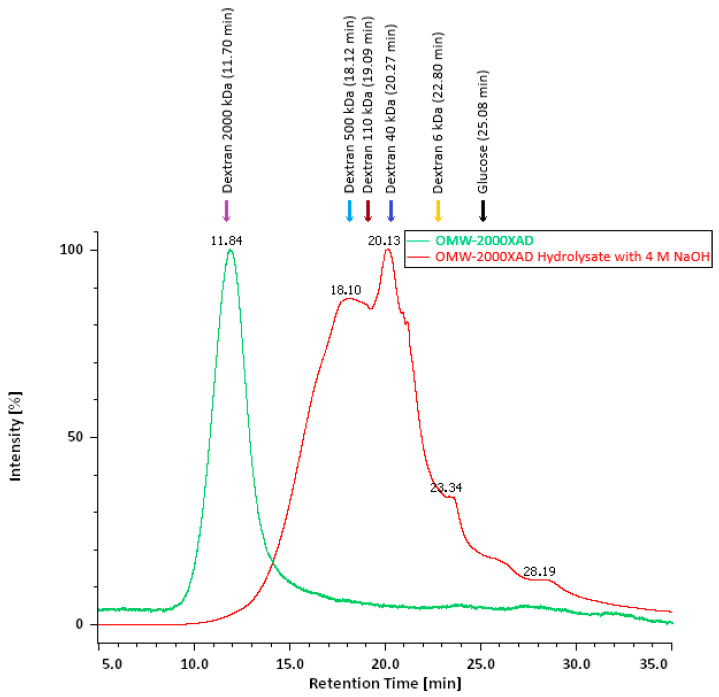
Gel filtration chromatogram of the OMW-2000XAD complex polymer (t_r_ = 11.84 min) (in green) and its hydrolysate, with 4 M NaOH (in red), glucose (t_r_ = 25.08 min), and several dextrans of increasing molecular weights: 6 kDa (t_r_ = 22.80 min), 40 kDa (t_r_ = 20.27 min), 70 kDa (t_r_ = 19.57 min), 110 kDa (t_r_ = 19.09 min), 500 kDa (t_r_ = 18.12 min), and 2000 kDa (t_r_ = 11.70 min), marked with colored arrows.

OMW-2000XAD was analyzed via gel permeation chromatography and was shown to have a high molecular weight, indicating that it is a supramolecular complex rather than simply a mixture of phenolic compounds, polysaccharides, protein, and metals, as all these components co-elute at the same position, indicating that they are bound. As Figure 2 shows, the gel filtration chromatogram of the OMW-2000XAD complex has only one peak (t_r_ = 11.84 min), which overlaps with the Blue Dextran 2000 peak (t_r_ = 11.70 min). The approximate molecular weight of the OMW-2000XAD complex was estimated by analyzing the standard solutions of dextrans of known molecular weight as references and comparing their retention times with the result of OMW-2000XAD. It can be seen that the dextran-soluble polymer compounds are eluted from the column in descending order of molecular weight. The plot log, M_w_ vs. t_r_ (R^2^ = 0.9987), yielded an estimated molecular weight for OMW-2000XAD of 1960 kDa. This molecular weight is considerably higher than that of polymerin (2–500 kDa), as described by Capasso and co-workers [21], and the melanin- and humic acid-like polymer complex OMWW-ASP (5–190 kDa), which is mainly composed of protein, as reported by Khemakhem and co-workers [28].

### 3.3. FTIR Analysis of OMW-2000XAD

**Figure 3:**
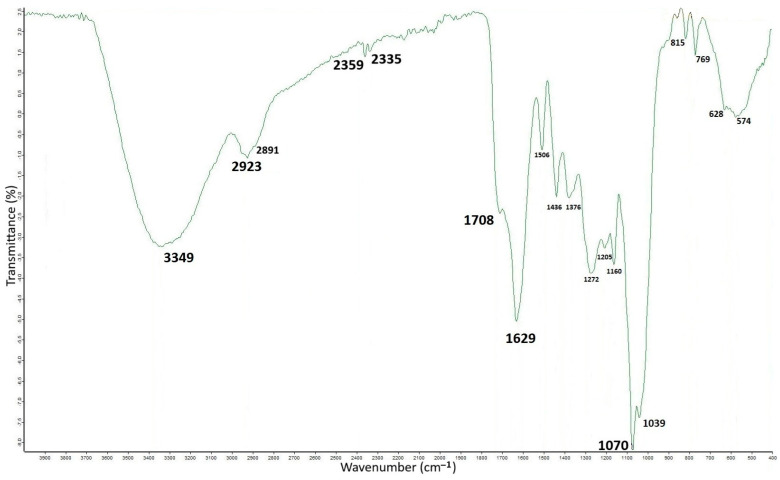
FTIR spectrum of OMW-2000XAD. The *x*-axis represents wavenumbers (cm^−1^) in the range of 4000–400 cm^−1^, while the *y*-axis indicates transmittance (%).

Figure 3 shows the mid-infrared (MIR) spectrum for the OMW-2000XAD polymer. FTIR spectroscopy is generally considered non-specific. In addition, OMW-2000XAD is a particularly complex substance, and, therefore, a strong spectral overlap makes it difficult to identify its components or functional groups and determine the chemical structure. The FTIR spectrum interpretation of OMW-2000XAD and a tentative assignment of the observed bands are based on the FTIR analyses of OMW fractions [21,28,36,40], as well as the complexes and conjugates of phenolic compounds, polysaccharides, and proteins. The data suggests the presence of multiple functional groups, indicating a structurally diverse compound.

The FTIR spectrum of OMW-2000XAD showed a very strong and broad stretching absorption signal centred at 3349 cm^−1^, which is common in aromatic and aliphatic hydroxyl groups (ν O-H) and characteristic of polysaccharides and phenolic compounds, whose functional groups form both intermolecular and intramolecular hydrogen bonds. These extensive molecular interactions lead to a wider band shape. Primary and secondary amine stretching absorption bands (ν N-H) of amino acids in the 3300–3500 cm^−1^ range are generally sharper and less intense than hydroxyl bands; thus, if they are present, they are not observed. The single-bond region (4000–2500 cm^−1^) also includes stretching vibrations of C-H bonds. Two much less intense bands than the preceding ones appear at 2923 cm^−1^ and 2880 cm^−1^, and they are indicative of aliphatic groups (-CH_2_ and -CH_3_, respectively), which are mainly typical of cellulose and hemicelluloses [41], as well as a polyphenolic network [42].

The double-bond region (2000–1500 cm^−1^) exhibits strong absorptions that suggest the presence of highly polar bonds attributable to ν C=O stretching vibrations of carbonyl groups in ketones, aldehydes, esters, or carboxylic acids at 1708 cm^−1^ and 1629 cm^−1^ of ν C=C stretching in conjugated systems, which are characteristic of biopolymers [43]. However, these bands may also be due to the amide I band for the protein secondary structure [44], since signals of proteins are observed in the range 1550–1700 cm^−1^. The MIR spectrum also showed absorption at 1506 cm^−1^, which is typical of C=C-C stretching in the aromatic structures of phenolics associated or linked to sugars by ester bonds [18,41], as well as C=N of secondary amides [36]. The fingerprint region (1500–500 cm^−1^) contains a complex absorption pattern due to overlapping peaks. It includes two moderate peaks at 1436 cm^−1^ and 1376 cm^−1^, which are typically related to C=O and C=C stretching vibrations, as well as the O-H, C-O, COO^-^, and C-H bending modes of methylene [28]. The bands detected at 1272 cm^−1^ and 1205 cm^−1^ can be identified as stretching vibrations ν (C-O-C) of phenolic moieties and O-H deformations of carboxyl groups. The group of strong absorptions at 1160 cm^−1^, 1070 cm^−1^, and 1039 cm^−1^ can be assigned to the C-O stretching vibration of phenolic compounds, C-NH_2_ stretching of basic amino acids, the alcoholic groups of polysaccharides, and the inorganic fraction of OMW-2000XAD [41]. Finally, the weak triad of peaks at 856 cm^−1^, 815 cm^−1^, and 769 cm^−1^ corresponds to out-of-plane (δ Aryl-H) bending vibrations and further supports the presence of aromatic compounds and suggests C-C bond formation due to polymerization [45] because, normally, they are much stronger bands.

### 3.4. NMR Analysis of OMW-2000 XAD

**Figure 4:**
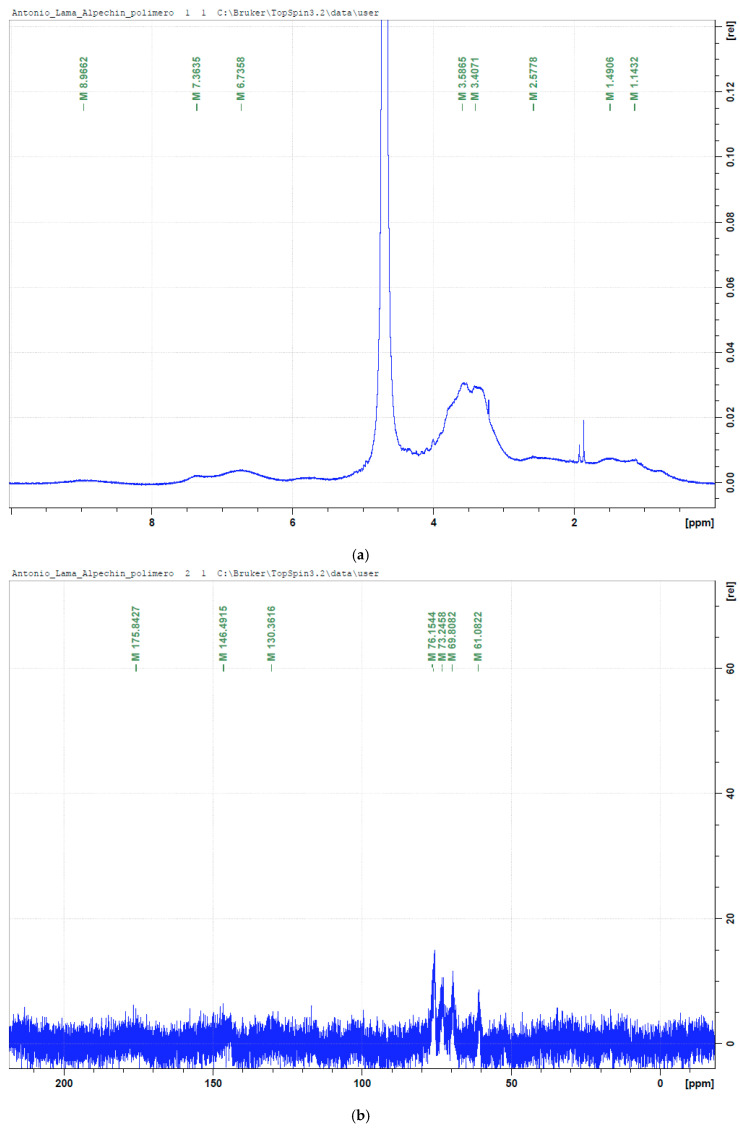

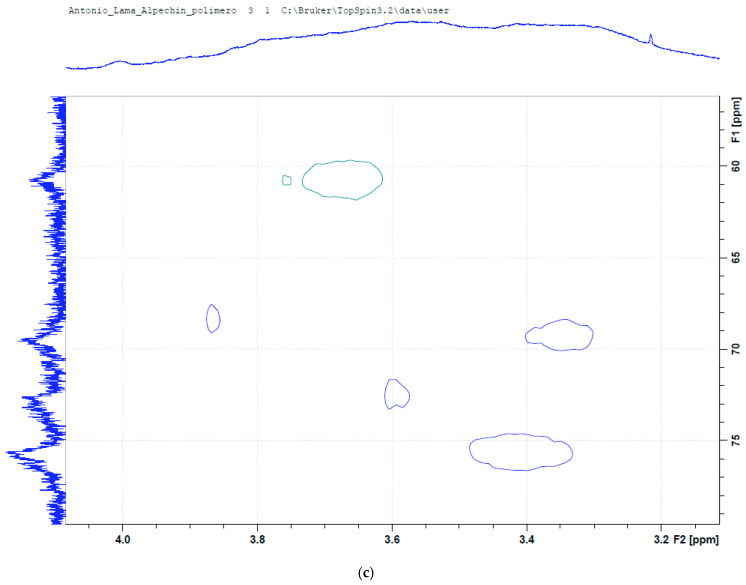
(**a**) ^1^H-NMR spectrum of OMW-2000XAD. (**b**) ^13^C-NMR spectrum of OMW-2000XAD. (**c**) ^1^H/^13^C-HSQC spectrum of OMW-2000XAD.

The NMR spectra were difficult to interpret and provided limited structural information with respect to the structure of OMW-2000XAD due to poor resolution and weak signals; in the ^1^H-NMR spectrum, the only notable signals observed were between 3 and 4 ppm and were very probably attributable to H_2–6_-CO protons of polysaccharides and groups such as -OCH_3_ [46] (the high content of glucose suggests that it could be a hemicellulose-rich complex). They were also observed between 6 and 8 ppm (signals at 6.7 and 7.4 ppm corresponding to aromatic rings protons of phenolic compounds) (Figure 4a) [47], and all of them were very broad. This may be attributed to protein signals [28]. On the other hand, the presence of anomeric protons could be masked by the dominant water peak at around 4.5–5 ppm. In the ^13^C-NMR spectrum (Figure 4b), signals are mainly observed at 60–70 ppm (chemical shifts of C2-C6), which confirms the presence of the HC-O of polysaccharides and is corroborated by the HSQC experiment (Figure 4c) and correlations, ^1^H/^13^C, observed in it (cross peaks at 3.35/69.4 ppm; 3.40/75.5 ppm; 3.59/72.5 ppm; 3.67/60.8 ppm, and 3.87/68.5 ppm). However, chemical shifts corresponding to aryl carbons (110–145 ppm), O-aryl carbons (145–165 ppm), and carboxyl carbons (165–190 ppm), as well as the assignment of phenolic and amide functional groups, were not observed or they were very weak (130.4, 146.5, and 175.8 ppm). Despite the use of high-field (500 MHz) equipment, the low solubility of OMW-2000XAD in water prevented the acquisition of high-quality spectra (spectra were recorded in a volume of 600 µL of water, and 50 mg of OMW-2000XAD did not dissolve completely). Attempts to improve the NMR spectra were unsuccessful due to the poor insolubility of the complex in most deuterated solvents. DMSO was tested without success. No significant improvement in spectral quality was achieved. Therefore, the structural information provided by NMR was inconclusive. This limitation underscores the need for future fractionation and more advanced solubility strategies to enable meaningful structural elucidation.

### 3.5. Mass Spectrometry for the Qualitative Analysis of Compounds in OMW-2000 XAD Hydrolysates

OMW-2000XAD was subjected to acid and alkali hydrolysis. These treatments were applied to disrupt the phenolic structure and linkages between this material and polysaccharides and proteins in order to identify the individual phenolic compounds via mass spectrometry. Figure 2 (GPC chromatogram) shows a clear decrease in the molecular weight of OMW-2000XAD after hydrolytic degradation. A significant shift in the retention time is observed from 11.84 min to 18.10–20.13 min.

**Figure 5:**
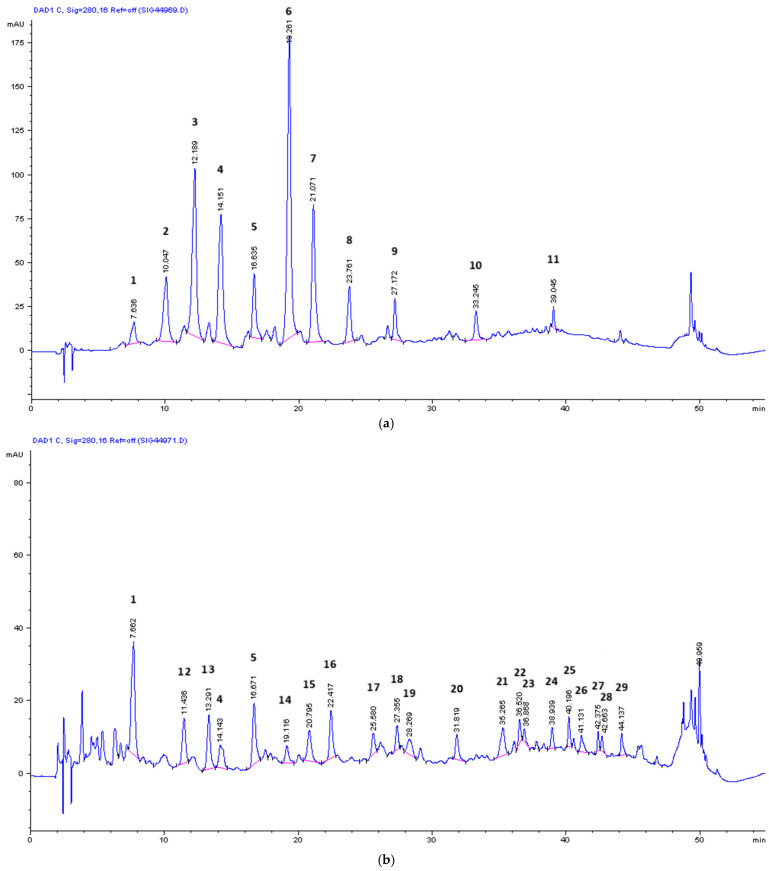
(**a**) HPLC chromatogram at 280 nm of the hydrolysate, with 4 M NaOH of OMW-2000XAD. A tentative identification of the peaks via ESI-Qq-TOF mass spectrometry can be seen in Table 3. (**b**) HPLC chromatogram at 280 nm of the hydrolysate, with 6 M HCl of OMW-2000XAD. A tentative identification of the peaks via ESI-Qq-TOF mass spectrometry can be seen in Table 3.

The phenolic profile of each of the hydrolysates was analyzed using LC-MS (ESI-Qq-TOF). The chromatograms obtained through this analysis are shown in Figure 5a,b. On the other hand, the compounds tentatively identified are presented in Table 3, along with the *m*/*z* of each peak [M-H]^-^, as well as the resulting fragments for that respective *m*/*z*, in addition to other information. In Figure 5a,b, the 29 most prominent peaks are indicated. Although some of the usual phenols from OMW are detected, primarily tyrosol, phenolic acids (caffeic, *p*-coumaric, and ferulic), and some elenolic acid derivatives, the remaining peaks could not be assigned to any known phenolic compound that was originally present in OMW or any other olive-derived product, despite an extensive literature review. In other cases, the fragments of the base peak do not match those reported in the literature. The authors hypothesize that these unknown compounds arise from phenolics that have been transformed because many of the masses found can be associated with the fragmentation of other known phenolic compounds derived from OMW. This hypothesis is based on the fact that the spectra of hydrolysates (mainly with NaOH) also show some masses and fragments that are signs of compounds resulting from the oxidation and the polymerization of phenols, such as covalent dimers, trimers, or tetramers of caffeic acid (t_r_ = 20.08 min, *m*/*z* 313.0741, 269.0867, and 179.0358 or 147.0113; (AH)_2_, C–O type or C-C type dimers), *p*-coumaric acid (t_r_ = 34.54 min, *m*/*z* 651.1514, 325.0703, 281.0802 and 237.0911; 4AH–4H, tetramer), dihydrocaffeic acid (t_r_ = 35.67 min, *m*/*z* 495.1769; [2AH + A] − 2H), and hydroxytyrosol (t_r_ = 26.11 min, *m*/*z* 455.1347, 303.0822; [2AH + A] − 2H) [48].

### 3.6. Antioxidant Capacity of OMW-2000XAD

The overall antioxidant capacity of OMW-2000XAD was evaluated using several antioxidant assays, each representing different antioxidant mechanisms. TPC via Folin–Ciocalteu reagent, TEAC (based on the inhibition of the ABTS^•+^ radical cation), FRAP, and DPPH are assays conducted via electron-transfer reactions that can be used to measure the reducing and radical scavenging capacity, while the ORAC assay involves hydrogen atom transfer reactions and can be used to quantify the peroxyl radical scavenging capacity. OMW-2000XAD turned out to be an effective free radical scavenger. The total antioxidant capacities of OMW-2000XAD ranged from 701 to 3783 µmol Trolox equivalent/g, as assessed using the TEAC, DPPH, FRAP, and ORAC methods (Table 4). This table also includes, for comparison purposes, the values of hydroxytyrosol (HT) and caffeic acid (CA), which are two of the best antioxidant phenolic compounds found in OMW. When values are expressed as a function of the calculated molecular weight (1960 kDa), the antioxidant capacity of OMW-2000XAD is thousands of times higher than those of HT and CA (for example, OMW-2000XAD has a value of 4976 mol Trolox equivalent/mol in TEAC, while HT and CA have only 1.7 and 1.9 mol Trolox equivalent/mol, respectively). It may reasonably be expected that the high phenolic content of OMW-2000XAD appears to be the key factor in determining its raised antioxidant capacity. However, in addition to this, the polymeric character of the complex, which was dialyzed free of unbound compounds, must undoubtedly be considered. Some authors have suggested that the polymerization of antioxidant phenolic compounds can reproduce and release -OH groups in the reaction products [57], which are evidently associated with a higher radical scavenging capacity than that of monomeric compounds, which aligns with the hypothesis that antioxidant capacity is proportional to the degree of polymerization and with the presence of many aromatic structures and oxidizable functional groups [25].

In comparison with other polymeric phenolic fractions from plant extracts, for example, OMW-2000XAD has an antioxidant capacity, as measured via the TEAC method, that is approx. 2- to 2.5-fold higher than that of grape stem extracts (>10 kDa) [58] and mango seed kernel extracts [59] (expressed as mmol Trolox equivalent/g), which are both rich in tannins and are also characterized by their ability to form strong complexes with macromolecules, such as protein and polysaccharides, among others. Although no specific data are provided on the molecular weights of these extracts, the results denote the potent antioxidant capacity of the polymeric phenolic fractions and that a clear correlation exists between the antioxidant capacity and the high molecular weight. When OMW-2000XAD is compared to a reference tannin, such as tannic acid, both show a similar value of ABTS^•+^ radical scavenging capacity (2.5 versus 3.1 mmol Trolox equivalent/g, respectively) [59]; however, this comparison does not take into account that the molecular weight of OMW-2000XAD is much higher than that of tannic acid (1960 kDa versus 1.7 kDa).

**Table 4:** Antioxidant capacity (µmol Trolox equivalent/g) and total phenolic content (TPC, µmol gallic acid equivalent/g) of the OMW-2000XAD complex. As a comparison, the values of major olive antioxidant phenolic compounds have been included.

Assay	OMW-2000XAD	Hydroxytyrosol	Caffeic Acid
TEAC	2539 ± 38 ^1^/(4976) ^2^	11,322/(1.7)	10,700/(1.9)
DPPH	701 ^3^ ± 4/(1374)	8784 ^3^/(1.4)	7148 ^3^/(1.3)
FRAP	1038 ± 35/(2035)	5550/(0.9)	Not found
ORAC	3783 ± 76/(7415)	40,405/(6.2)	Not found
TPC	5277 ± 186/(10,343)	-	-

^1^ Standard error of the mean of three replicates (*n* = 3). ^2^ Results given in parentheses are expressed as mol Trolox equivalent/mol. ^3^ IC_50_ value. The values for hydroxytyrosol and caffeic acid were taken from Bermúdez-Oria and co-workers [60] and Rubio-Senent and co-workers [61].

### 3.7. Anti-Proliferative Activity of OMW-2000XAD

**Figure 6:**
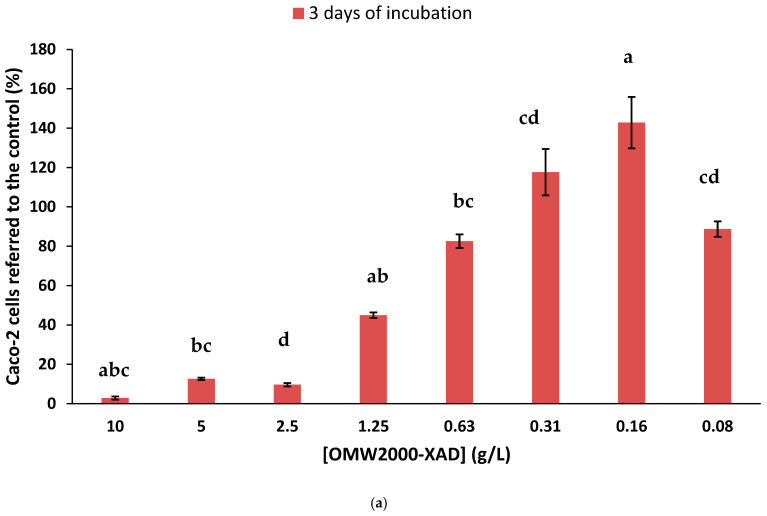

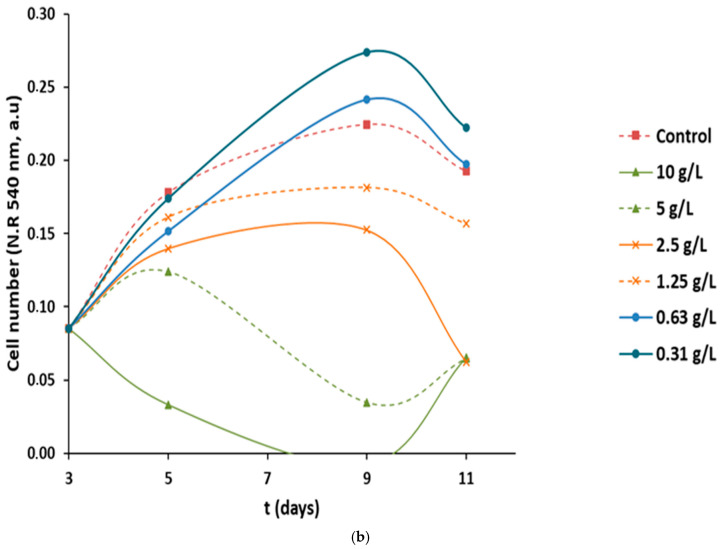
(**a**) Effect of OMW2000-XAD on Caco-2 cell proliferation at various concentrations (from 0.08 g/L to 10 g/L). Neutral red uptake was determined after 3 days of incubation. Data are the mean of six replicates ± the standard deviation. Different letters indicate statistically significant differences (data analyzed via a one-way ANOVA-LSD test at a significance level of *p* < 0.05). (**b**) Effect of OMW-2000XAD on proliferation of confluent Caco-2 cells. The cell number was estimated via the determination of neutral red uptake. Data are the mean of six replicates. Error bars and statistically significant differences are not shown for clarity.

The results of the treatment of Caco-2 cells with OMW-2000XAD (Figure 6a) indicate that it does not exert an antiproliferative effect. After 3 days of exposure to OMW2000-XAD, no significant reduction in cell growth was observed at the lower doses (0.08–0.63 g/L) compared to the control culture. However, at higher concentrations (1.25–10 g/L), OMW-2000XAD seems to reduce the growth of and kill Caco-2 cells. Unfortunately, we presume that this effect is not a consequence of the antiproliferative ability but is due to damage caused by toxicity, because when the effect of OMW-2000XAD on the cell proliferation of confluent Caco-2 cells was evaluated (Figure 6b), similar results were obtained. Caco-2 cells differentiate into an enterocyte-like phenotype when they are allowed to grow as confluent monolayers [62].

## 4. Conclusions

This study reports the successful isolation of a previously undescribed high-molecular weight phenolic polymer, OMW-2000XAD, from olive mill wastewater using a reproducible and straightforward chromatographic method. Although only OMW from the Picual variety was used, the method was successfully replicated with a batch from a different origin, suggesting good reproducibility. The complex has a high content of phenolic compounds and exhibits exceptional antioxidant capacity, making it a promising candidate for various environmental, food, and agricultural applications. Potential uses for OMW-2000XAD include its incorporation as an antioxidant additive in packaging materials, a remediation agent for contaminated water, a biopesticide, an antimicrobial compound, and a functional ingredient in biomaterials—among others—as suggested by the existing literature on plant-derived phenolic polymers. However, spectroscopic data suggest the absence of a regular repeating monomeric unit or moiety, and the available spectra—using currently accessible techniques—do not provide sufficient information to propose a plausible polymeric structure (linear, branched, or network polymer) or to determine the linkage patterns among phenolic compounds, polysaccharides, and proteins. This challenge is further compounded by the multicomponent nature and high molecular weight of OMW-2000XAD. In future work, the authors aim to fractionate OMW-2000XAD into lower-molecular weight components to facilitate structural characterization and linkage analysis, as well as to improve its biological activity while more thoroughly investigating its potential applications. It would also be interesting to study the production of OMW-2000XAD from OMW derived from other olive varieties to assess whether the procedure described herein can overcome one of the main limitations of biomass-based materials, compositional variability, which may significantly influence the complex’s bioactivity and applicability. Finally, this study highlights the use of a discarded olive oil industry byproduct effluent for extracting natural complex antioxidants, which aligns with current recommendations for reducing pollution from food processing. This approach contributes to both sustainability and environmental responsibility, which can be attractive to researchers and the general public.

## Figures and Tables

**Table 1:** Contents expressed in percentages of phenols, polysaccharides, and proteins, as well as the elemental chemical composition of the complex polymer OMW-2000XAD. Values are means of three replicates (independently prepared samples).

Organic Component	Element	% (g/100 g)
Phenols		89.8 ± 0.3 ^4^
Polysaccharides		16.1 ± 0.5
Protein		10.3 ± 0.1
	C ^1^	46.31 ± 0.19
	N	1.64 ± 0.01
	Na ^2^	0.03 ± 0.01
	Mg	0.13 ± 0.01
	P	0.03 ± 0.01
	S	0.75 ± 0.02
	K	0.73 ± 0.02
	Ca	0.34 ± 0.01
	Fe	0.03 ± 0.01
	Cu	0.03 ± 0.01
	Total metals ^3^	1.29

^1^ Determined by a CHN analyzer. ^2^ Determined by ICP-MS. ^3^ Excluding P and S. ^4^ Standard error of the mean of three replicates (*n* = 3).

**Table 2:** Sugar and amino acid compositions of OMW-2000XAD, as determined via GC and HPLC chromatography, respectively.

Sugar	% (Referred to Carbohydrate Content)	g/100 g OMW-2000XAD	Amino Acid	% (Referred to Protein Content)	g/100 g OMW-2000XAD
Glucose	75.8	12.25 ± 0.11 ^1^	Cysteine (Cys)	16.6	1.71 ± 0.04
Arabinose	6.8	1.11 ± 0.02	Glutamic (Glu)	12.4	1.27 ± 0.03
Rhamnose	5.6	0.91 ± 0.02	Aspartic (Asp)	10.9	1.13 ± 0.01
Galactose	5.0	0.76 ± 0.01	Glycine (Gly)	10.5	1.08 ± 0.02
Mannose	3.7	0.58 ± 0.01	Serine (Ser)	7.3	0.75 ± 0.01
Xylose	3.1	0.49 ± 0.01	Tryptophan (Trp)	6.9	0.71 ± 0.05
			Alanine (Ala)	5.6	0.58 ± 0.02
			Leucine (Leu)	5.5	0.57 ± 0.01
			Threonine (Thr)	5.3	0.54 ± 0.03
			Arginine (Arg)	4.4	0.45 ± 0.01
			Phenylalanine (Phe)	3.9	0.40 ± 0.02
			Valine (Val)	3.7	0.38 ± 0.01
			Isoleucine (Ile)	2.8	0.28 ± 0.01
			Tyrosine (Tyr)	1.9	0.20 ± 0.01
			Histidine (His)	1.1	0.11 ± 0.01
			Lysine (Lys)	0.6	0.04 ± 0.01
			Methionine (Met)	0.3	0.06 ± 0.01
			Proline (Pro)	0.3	0.03 ± 0.01
Total	100	16.1	Total	100	10.3

^1^ Standard error of the mean of three replicates (*n* = 3).

**Table 3:** Main molecular ions and their fragments detected in the hydrolysates, with 4 M NaOH and 6 M HCl of OMW-2000XAD identified via HPLC-DAD-ESI-Qq-TOF (negative mode). The identification of phenolic compounds was tentatively confirmed via comparison with the data reported in the literature.

t_r_ (min)	[M-H]^-^ *m*/*z*	Fragment Ions *m*/*z*	UV_max_ (nm)	Compound	Hydrolysate 4 M NaOH	Hydrolysate 6 M HCl	Ref.
7.64	331.0026 (4.5)	219.0523 (6.8) 153.0204 (100) 109.0287 (59.2)	207, 217, 260, 294	1 *—Unknown	+	+	-
10.05	137.0252 (100)	-	228, 280	2—Tyrosol	+	-	[49]
11.44	293.1230 (5.9)	213.0778 (18.0) 137.0253 (100)	224, 283, 311	12—Unknown	-	+	[50]
12.19	151.0412 (100)	109.0289 (3.8)	200, 230, 276, 306	3—Vanillin	+	-	[51]
13.29	213.0774 (44.8)	151.0406 (100)	207, 228, 275, 306	13—Elenolic acid derivative	-	+	[5]
14.15	179.0361 (68.1)	135.0462 (100)	218, 323	4—Caffeic acid	+	+	[48]
16.64	317.0292 (20.6)	165.0200 (100) 121.0298 (38.5)	209	5—Unknown	+	+	-
19.12	221.0388 (4.4)	215.0927 (12.3) 187.0176 (100) 135.0463 (75.9)	200, 281	14—Unknown	-	+	-
19.26	163.0406 (21.8)	119.0507 (100)	227, 310	6—*p*-Coumaric acid	+	-	[52]
20.08	313.0741 (11.7)	269.0867 (45.6) 179.0358 (100) 147.0113 (13.0)	310	Caffeic acid (dimers C-C and C-O type)	+	-	[48]
20.80	341.1203 (58.9)	179.0327 (100) 135.0076 (1.5)	218, 262, 296	15—Caffeoyl hexoside	-	+	[53]
21.07	193.0490 (54.0)	178.0285 (91.0) 134.0379 (100)	218, 238, 323	7—t-Ferulic acid	+	-	[52]
22.42	225.0756 (100)	-	-	16—Desoxyelenolic acid	-	+	[5]
23.76	359.1110 (5.0)	203.0354 (100) 159.0460 (84.0)	232, 333	8—Syringic acid hexoside	+	-	[54]
25.58	491.0932 (3.5)	397.0186 (5.3) 279.0403 (26.9) 203.0289 (37.5) 170.9856 (100)	208, 270	17—Unknown	-	+	-
26.11	455.1347 (45.9)	303.0822 (4.2)	-	Hydroxytyrosol [(2AH + A) − 2H]	-	+	[48]
27.17	325.0701 (11.3)	283.0618 (14.9) 197.0821 (22.9) 153.0928 (100)	267	9—Unknown	+	-	-
27.35	365.1202 (4.9)	229.1080 (100) 199.0902 (48.2) 153.0931 (92.6) 123.0802 (35.6)	-	18—Dimethyl acetal of oleacein	-	+	[55]
28.27	221.0780 (100)	161.0246 (16.0)	205	19—Unknown	-	+	-
31.82	411.0476 (2.4)	329.0993 (35.6) 257.0339 (100) 189.0515 (17.2) 147.0088 (3.4)	205, 251	20—Unknown	-	+	-
33.25	439.1962 (8.5)	355.0806 (44.0) 325.0703 (79.5) 281.0812 (24.7) 159.0461 (100)	229	10—Unknown	+	-	-
34.54	651.1514 (2.9)	325.0699 (100) 281.0811 (19.5) 237.0917 (19.2)	-	*p*-Coumaric acid (tetramer)	+	-	[48]
35.27	569.0031 (4.2)	411.1591 (10.2) 267.0866 (30.7) 235.0603 (100) 197.0599 (8.4)	230, 316	21—Unknown	-	+	-
35.67	495.1771 (25.7)	-	-	Dihydrocaffeic acid [(2AH + A) − 2H]	+	-	[48]
36.52	437.1101 (3.7)	393.0810 (7.2) 301.0697 (44.2) 249.0753 (100) 225.0541 (29.9)	226, 280	22—Unknown	-	+	-
36.87	949.6636 (2.9)	549.1617 (100) 473.0985 (2.7) 381.0401 (4.3) 287.0343 (3.0) 257.0800 (9.3) 225.0548 (2.9)	229, 316	23—Unknown	-	+	-
38.94	229.1034 (3.4)	177.0521 (100) 141.0749 (10.4) 123.0090 (1.0)	202, 291	24—3,4-Dihydroelenolic acid (demethylated)	-	+	[56]
39.05	519.3151 (4.6)	351.0863 (100) 295.0261 (8.4)	230, 310	11—Unknown	+	-	-
40.20	437.1204 (6.5)	377.0486 (2.5) 257.0807 (60.0) 213.0917 (36.3) 181.0657 (100)	227	25—Unknown	-	+	-
41.13	494.0700 (7.8)	473.0988 (7.8) 347.0385 (19.2) 263.0887 (100) 219.0811 (18.6) 171.0434 (15.6) 113.0862 (4.0)	240, 280, 303	26—Unknown	-	+	-
42.38	507.0619 (23.9)	383.0416 (10.7) 337.0721 (7.9) 249.0702 (24.3) 205.0826 (100)	-	27 -Unknown	-	+	-
42.66	257.0814 (38.8)	225.0556 (2.5) 213.0905 (3.8) 181.0659 (100) 153.0716 (8.5)	225	28—Hydroxyelenoic acid	-	+	[50]
44.14	265.1498 (2.4)	213.0095 (100)	208, 231	29—Xanthonic acid	-	+	[50]

Intensities relative to the base peak are given in parentheses. * Peak number assigned in the chromatograms from Figure 5a,b.

## Data Availability

Data is contained within the article.
